# Both *SEPT2 *and *MLL *are down-regulated in *MLL-SEPT2 *therapy-related myeloid neoplasia

**DOI:** 10.1186/1471-2407-9-147

**Published:** 2009-05-15

**Authors:** Nuno Cerveira, Joana Santos, Susana Bizarro, Vera Costa, Franclim R Ribeiro, Susana Lisboa, Cecília Correia, Lurdes Torres, Joana Vieira, Simone Snijder, José M Mariz, Lucília Norton, Clemens H Mellink, Arjan Buijs, Manuel R Teixeira

**Affiliations:** 1 Department of Genetics, Portuguese Oncology Institute, Porto, Portugal; 2 Department of Clinical Genetics, Academic Medical Center, Amsterdam, The Netherlands; 3 Department of Hemato-Oncology, Portuguese Oncology Institute, Porto, Portugal; 4 Department of Pediatrics, Portuguese Oncology Institute, Porto, Portugal; 5 Department of Medical Genetics, University Medical Center, Utrecht, The Netherlands; 6 Abel Salazar Biomedical Sciences Institute (ICBAS), Porto, Portugal

## Abstract

**Background:**

A relevant role of septins in leukemogenesis has been uncovered by their involvement as fusion partners in *MLL*-related leukemia. Recently, we have established the *MLL-SEPT2 *gene fusion as the molecular abnormality subjacent to the translocation t(2;11)(q37;q23) in therapy-related acute myeloid leukemia. In this work we quantified *MLL *and *SEPT2 *gene expression in 58 acute myeloid leukemia patients selected to represent the major AML genetic subgroups, as well as in all three cases of *MLL-SEPT2*-associated myeloid neoplasms so far described in the literature.

**Methods:**

Cytogenetics, fluorescence in situ hybridization (FISH) and molecular studies (RT-PCR, qRT-PCR and qMSP) were used to characterize 58 acute myeloid leukemia patients (AML) at diagnosis selected to represent the major AML genetic subgroups: *CBFB-MYH11 *(n = 13), *PML-RARA *(n = 12); *RUNX1-RUNX1T1 *(n = 12), normal karyotype (n = 11), and *MLL *gene fusions other than *MLL-SEPT2 *(n = 10). We also studied all three *MLL-SEPT2 *myeloid neoplasia cases reported in the literature, namely two AML patients and a t-MDS patient.

**Results:**

When compared with normal controls, we found a 12.8-fold reduction of wild-type *SEPT2 *and *MLL-SEPT2 *combined expression in cases with the *MLL-SEPT2 *gene fusion (p = 0.007), which is accompanied by a 12.4-fold down-regulation of wild-type *MLL *and *MLL-SEPT2 *combined expression (p = 0.028). The down-regulation of *SEPT2 *in *MLL-SEPT2 *myeloid neoplasias was statistically significant when compared with all other leukemia genetic subgroups (including those with other *MLL *gene fusions). In addition, *MLL *expression was also down-regulated in the group of *MLL *fusions other than *MLL-SEPT2*, when compared with the normal control group (p = 0.023)

**Conclusion:**

We found a significant down-regulation of both *SEPT2 *and *MLL *in *MLL-SEPT2 *myeloid neoplasias. In addition, we also found that *MLL *is under-expressed in AML patients with *MLL *fusions other than *MLL-SEPT2*.

## Background

Septins comprise an evolutionarily conserved family of GTP-binding proteins that are found primarily in fungi and animals [1]. In humans, 14 septin genes have been characterized to date (*SEPT1 *to *SEPT14*). All septin transcripts contain multiple translation initiation sites and are alternatively spliced, giving origin to multiple septin isoforms, some of which are tissue specific [1]. Although the precise functions of septins remain unclear, current data suggest that they coordinate changes in cytoskeletal and membrane organization by acting as scaffolds that recruit factors to precise sites in a cell and/or as barriers that segregate membrane areas into discrete domains [1,2]. For instance, the human SEPT2 associates with SEPT6 and SEPT7 to form an hexamer complex that is the core unit for generation of septin filaments associated with the contractile ring in dividing cells, being therefore essential for proper cytokinesis and chromosome segregation [2,3].

Septins have been reported to be deregulated in various human diseases, including cancer [4]. A relevant role of septins in leukemogenesis has been uncovered by their involvement as fusion partners in *MLL*-related leukemia. We have established the *MLL-SEPT2 *gene fusion as the molecular abnormality subjacent to the translocation t(2;11)(q37;q23) in therapy-related acute myeloid leukemia (t-AML) [5]. Subsequently, van Binsbergen et al [6] identified a second *MLL-SEPT2 *fusion variant in a patient with t-AML and we have recently uncovered a third *MLL-SEPT2 *alternative fusion variant in a case of therapy-related myelodysplastic syndrome (t-MDS) [7,8]. Four other septin family genes (*SEPT5*, *SEPT6*, *SEPT9 *and *SEPT11*) had previously been identified as *MLL *fusion partners in leukemia, making the septins the protein family with more numbers involved in *MLL*-related leukemia [9-13], and suggesting that their involvement in leukemogenesis is not a chance event. This hypothesis is supported by the fact that all the reported *MLL*-septin fusions are in frame and the breakpoints are found at the very 5' end of known septin open reading frames [5,9-13]. In this work we show evidence that the fusion of *MLL *with *SEPT2 *is associated with down-regulation of both *SEPT2 *and *MLL *expression in t-AML/t-MDS.

## Methods

### Patient samples

We studied 58 acute myeloid leukemia patients (AML) at diagnosis selected to represent the major AML genetic subgroups, including 13 cases with a *CBFB-MYH11 *rearrangement, 12 cases with a *PML-RARA *rearrangement, 12 cases with a *RUNX1-RUNX1T1 *rearrangement, 11 cases with normal karyotype, and 10 cases with rearrangements of the *MLL *gene other than *MLL-SEPT2 *[see Additional file 1]. We also studied all three *MLL-SEPT2 *patients reported in the literature, namely two with t-AML (patients 59 and 60) and the third with t-MDS (patient 61), which were the primary targets of our investigation [see Additional file 1] [5-8]. All but patients 60 and 61 were treated at the Portuguese Oncology Institute, Porto, Portugal, and bone marrow samples were obtained to perform cytogenetic and molecular studies. Patients 60 and 61 were treated in the Netherlands at the St. Antonius Hospital, Nieuwegein [6] and the Amsterdam Academic Medical Center [7], respectively, from whom cDNA was obtained.

As a control group we studied bone marrow samples obtained from ten individuals studied for the purpose to rule out a hematological disease.

This study was approved by the Portuguese Oncology Institute ethic committee, and informed consent was obtained from all patients.

### Chromosome banding and molecular cytogenetics

The diagnostic bone marrow samples of the Portuguese patients (cases 1 to 59) were cultured for 24 hours in RPMI 1640 medium with GlutaMAX-I (Invitrogen, London, UK) supplemented with 20% fetal bovine serum (Invitrogen, London, UK). Chromosome preparations were made by standard methods and banded by trypsin-Leishman. Karyotypes were described according to the International System for Human Cytogenetic Nomenclature [14].

Whenever appropriate, fluorescence in situ hybridization (FISH) analysis for specific fusion genes or rearrangements was performed using dual-color, break-apart or dual-fusion, probes (Vysis, Downers Grove, USA).

Chromosome banding and molecular cytogenetic analyses of patients 60 and 61 were described previously [6,7].

### RNA extraction and cDNA synthesis

Total RNA was extracted from the diagnostic bone marrow sample of patients 1 to 59 and controls using 1 ml of Tripure isolation reagent (Roche Diagnostics, Indianapolis, USA) and quantified in a NanoDrop ND-100 spectrophotometer (NanoDrop Technologies, Wilmington, USA). For cDNA synthesis, 1 μg of total RNA was subjected to reverse transcription with random hexamers using the Superscript III First-Strand Synthesis System for RT-PCR (Invitrogen, Carlsbad, USA), according to the manufacturer's instructions. The final cDNA was diluted with 30 μl of H_2_O. cDNA quantity and quality were assessed in a NanoDrop ND-100 spectrophotometer (NanoDrop Technologies, Wilmington, USA).

### Qualitative Reverse-Transcription Polymerase Chain Reaction (RT-PCR)

RT-PCR assays for detection of the fusion transcripts *RUNX1-RUNX1T1*, *CBFB-MYH11*, and *PML-RARA *were performed on the diagnostic samples according to the BIOMED-1 protocol [15]. The primers and PCR reaction conditions for the detection of rearrangements involving the *MLL *gene were previously published [5,10,16-19].

### Quantitative Real-Time Polymerase Chain Reaction (qRT-PCR)

We have evaluated the mRNA expression of *MLL *and *SEPT2 *genes by qRT-PCR on an ABI PRISM 7000 Sequence Detection System (Applied Biosystems, Foster City, USA). Primers and probes for *MLL *and *SEPT2 *were derived from the published mRNA sequences of *SEPT2 *and *MLL *(GenBank accession nos. NM_001008491.1 and NM_005933.2, respectively), and designed with Primer Express 2.0 (Applied BioSystems) and purchased from Metabion (Metabion, Martinsried, Deutschland) [see Additional file 2]. Primers and probes for the *ABL1 *gene (GenBank accession no. NM_005157), used as endogenous control, were previously described and approved for qRT-PCR based diagnosis and minimal residual disease detection in leukaemic patients, due to be similarly expressed in normal and diagnostic samples as well as within normal samples [see Additional file 2] [20,21]. All primers and probes were designed outside of *MLL *and *SEPT2 *breakpoint cluster regions in exons 4–5 and exons 3–4, respectively. To determine the relative expression levels of the target gene in each sample, the relative amount of the target gene was calibrated to the relative amount of the internal reference gene and expressed in terms of ratios between the target and the reference that were then multiplied by 100 for easier tabulation (target gene/*ABL1 *×100). PCR reactions were performed in a 25 μl volume containing 5 μl of synthesized cDNA, 12.5 μl of TaqMan universal PCR master mix, 0.3 μM of each primer and 0.2 μM of each probe. PCR was performed in separate wells for each primer/probe set and each sample was run in triplicate. PCR parameters were as follows: 50°C for 2 min., 95°C for 10 min., followed by 50 cycles at 95°C for 15 s. and 60°C for 1 min. Each plate included non-template controls and serial dilutions of a strongly expressing sample (*MLL *or *SEPT2*) to construct the standard curves.

### Bisulfite treatment

Sodium bisulfite conversion of unmethylated (but not methylated) cytosine residues to uracil in a sample of genomic DNA obtained from *MLL-SEPT2 *case 59 (DNA from *MLL-SEPT2 *cases 60 and 61 was not available) and three normal controls was performed as previously described [22]. Briefly, 500 ng of genomic DNA was denatured with 0.3 M NaOH in a total volume of 21 μl for 20 min. at 50°C. A volume of 450 μl freshly prepared bisulfite solution (2.5 M sodium bisulfite, 125 mM hydroquinone, and 0.2 M NaOH) was added to each denaturation reaction, and the mixture was incubated at 70°C for 3 hours in the dark. The resulting bisulfite-converted DNA was then purified by using Wizard DNA purification resin (Wizard DNA Clean-Up System; Promega, Madison, USA) according to the manufacturer's instructions and eluted in 45 μl of water preheated at 70°C. The eluted DNA was denatured in 0.3 M NaOH for 10 min. at room temperature. Finally, the bisulfite converted and denatured genomic DNA was precipitated with 100% ethanol, dried, resuspended in 30 μl of water, and stored at -20°C.

### Quantitative Methylation-Specific Polymerase Chain Reaction (qMSP)

Due to the very low quantity of DNA available from *MLL-SEPT2 *case 59, only the *SEPT2 *gene could be analyzed by qMSP. The *SEPT2 *5'-CpG island was identified using the CpG Island Searcher http://cpgislands.com[23] and the *SEPT2 *genomic sequence (GenBank accession no. NT_005416.12). That CpG island was found to encompass the predicted *SEPT2 *promoter region using PROSCAN 1.7 [24]. Primers and probe for the *SEPT2 *5'-CpG island were designed with Methyl Primer Express 1.0 (Applied BioSystems, Foster City, USA), and selected to specifically amplify fully methylated bisulfite-converted DNA [see Additional file 2]. Primers and probe for the internal reference gene, *ACTB *(GenBank accession no. NM_001101), were described previously [25] and were designed to amplify and detect a region of the gene that is devoid of CpG nucleotides to normalize for DNA input in each sample [see Additional file 2]. qMSP of the chemically modified DNA was performed in an ABI PRISM 7000 Sequence Detection System (Applied Biosystems, Foster City, USA), as previously described [26]. In brief, fluorescence based real-time PCR assays were carried out in a reaction volume of 20 μL, consisting of 16.6 mM ammonium sulfate, 67 mM trizma preset, 6.7 mM MgCl_2_, 10 mM mercaptoethanol, 0.1% DMSO, 200 μM each of dATP, dCTP, dGTP, and dTTP, 600 nM of each primer, 0.4 μL of Rox dye, 200 nM of probe, 1 unit of platinum Taq polymerase (Invitrogen, Carlsbad, USA), and 2 μl of bisulfite-modified DNA as a template. PCR was performed in separate wells for each primer/probe set and each sample was run in triplicate. PCR was performed under the following conditions: 95°C for 2 min., followed by 45 cycles of 95°C for 15 s. and 60°C for 1 min.

To ensure the specificity of the analysis, each 96-well PCR plate had wells that contained completely methylated DNA at all CpGs (positive control – CpGenome Universal Methylated DNA, Chemicon Europe, Hampshire, UK), a completely unmethylated DNA (negative control – CpGenome Universal Unmethylated DNA, Chemicon Europe, Hampshire, UK), and multiple water blanks (contamination control). To determine the relative levels of methylated promoter DNA in each sample, we used serial dilutions of the positive control DNA to construct the calibration curve. The values obtained (mean quantity) for each target gene were divided by the respective values of the internal reference gene (*ACTB*). The ratio thus generated, which constitutes an index of the percentage of input copies of DNA that are fully methylated at the primer- and probe-binding sites, was then multiplied by 100 for easier tabulation (methylation level = target gene/reference gene × 100).

### Statistical analyses

Normalized expression values for *MLL *and *SEPT2 *were compared among the different sample groups using the non-parametric Kruskal-Wallis H and Mann-Whitney U tests. The correlation between *MLL *and *SEPT2 *expression values within each group was assessed using Pearson's test. All analyses were performed using SPSS version 15.0 (SPSS, Chicago, USA).

## Results

In all cases, RNA and/or cDNA quantity and quality was evaluated and was found to be appropriate for expression studies. The 260/280 and 260/230 absorbance ratios for RNA samples were in the range of 1.8–2.2. For the cDNA samples, the 260/280 and 260/230 sample absorbance ratios were in the range of 1.6–2.0 and 1.8–2.2, respectively.

**Figure 1:**
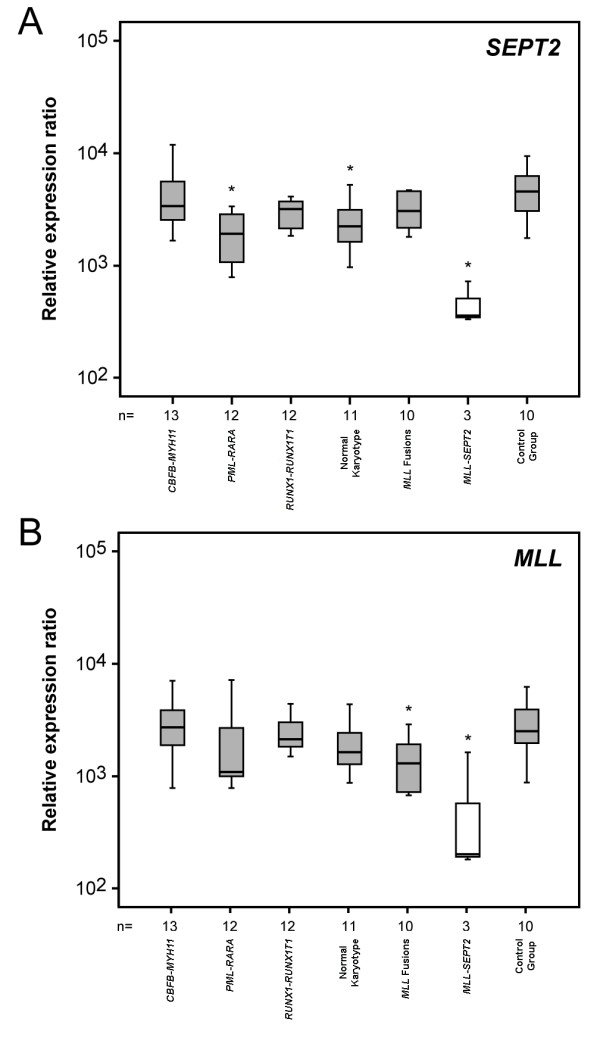
**Box-plots of normalized expression values for *SEPT2 *(A) and *MLL *(B) in subgroups of myeloid malignancies and control samples**. Asterisks denote significant differences when compared with the normal control group. The combined expression of *MLL-SEPT2 *and *MLL *or *SEPT2 *was significantly lower in *MLL-SEPT2 *patients compared to all other groups (see Table 1 for significance values).

Normalized expression levels for *MLL *and *SEPT2 *within each sample group are depicted in Figure 1. Statistically significant differences were observed for the combined wild-type *SEPT2 *and *MLL-SEPT2 *expression in the *MLL-SEPT2 *cases when compared with the normal controls, showing a 12.8-fold lower median expression in the *MLL-SEPT2 *subset (p = 0.007). Furthermore, the combined wild-type *SEPT2 *and *MLL-SEPT2 *expression was significantly lower (5.4 to 9.4 fold) in the *MLL-SEPT2 *cases than in all other leukemia genetic subgroups (Table 1). The combined expression of wild-type *MLL *and *MLL-SEPT2 *was also significantly lower (12.4 fold; p = 0.028) in the *MLL-SEPT2 *myeloid neoplasias when compared with the normal controls, as well as with the *CBFB-MYH11 *and *RUNX1-RUNX1T1 *leukemia subgroups (13.4 and 10.5 fold, respectively). We next investigated whether DNA hypermethylation of the 5' *SEPT2 *region was contributing to the *SEPT2 *gene down-regulation, by examining the methylation status of the CpG island located upstream of the *SEPT2 *gene transcriptional initiation site [2225 base pair (bp) in length (-7457 to -9257)]. *SEPT2 *5' CpG island hypermethylation was detected in the positive control, but not in the *MLL-SEPT2 *case 59 or the normal controls.

**Table 1:** Normalized values for the expression of *MLL-SEPT2 *and *MLL *or *SEPT2 *in subgroups of hematologic malignacies and normal controls

		SEPT2 *+ *MLL-SEPT2				MLL *+ *MLL-SEPT2			
	
Genetic groups	n	Median (P25–P75)	Fold Change (a)	*P-value (a)*	*P-value (b)*	Median (P25–P75)	Fold Change (a)	*P-value (a)*	*P-value (b)*
*CBFB-MYH11*	13	3388 (2411–5634)	9.4	*0.004*	*Ns*	2692 (1698–5099)	13.4	*0.025*	*ns*
*PML-RARA*	12	1957 (1055–2904)	5.4	*0.004*	*0.001*	1083 (971–2698)	5.4	*ns*	*ns*
*RUNX1-RUNX1T1*	12	3187 (2100–3760)	8.9	*0.004*	*ns*	2107 (1765–3018)	10.5	*0.018*	*ns*
Normal Karyotype	11	2242 (1604–3424)	6.2	*0.005*	*0.010*	1621 (1012–2895)	8.1	*ns*	*ns*
*MLL *Fusions	10	3069 (2159–4600)	8.5	*0.007*	*ns*	1292 (708–2050)	6.4	*ns*	*0.023*
*MLL-SEPT2*	3	359 (333–724)	1.0	*-*	*0.007*	200 (180–1612)	1.0	*-*	*0.028*
Normal Control	10	4599 (2939–6365)	12.8	*0.007*	*-*	2494 (1749–3929)	12.4	*0.028*	*-*

(a) Comparison of each group with *MLL-SEPT2 *patients; (b) Comparison of each group with normal control group; (P25) percentile 25; (P75) percentile 75.

No statistically significant differences were observed for the wild-type *SEPT2 *expression between the non-*MLL-SEPT2 *leukemia subgroups and the normal controls, with the exception of the *PML-RARA *and "normal karyotype" leukemias that showed lower expression (Table 1). No statistically significant differences were observed for the wild-type *MLL *expression between the non-*MLL-SEPT2 *leukemia subgroups and the normal controls, with the exception of the significantly lower expression seen in the patient group with *MLL *fusions with other partners other than *SEPT2 *(q = 0,023).

## Discussion

Fusion oncogenes are generally thought to contribute to carcinogenesis by either causing over-expression of the 3' partner due to promoter swap or by originating a chimeric protein with new biochemical properties. Surprisingly, when compared with the normal controls, we found a 12.8-fold reduction of the combined *MLL-SEPT2 *and wild-type *SEPT2 *expression in myeloid neoplasias with the *MLL-SEPT2 *gene fusion, which is accompanied by 12.4-fold down-regulation of the combined *MLL-SEPT2 *and wild-type *MLL *expression. The down-regulation of *SEPT2 *in *MLL-SEPT2 *myeloid neoplasias was also statistically significant when compared with all other leukemia genetic subgroups (including those with other *MLL *gene fusions). It is conceivable that deregulation of *SEPT2 *expression can occur as a result of its fusion with *MLL *(for example by haplo-insufficiency), since the *MLL-SEPT2 *gene is under the control of the *MLL *promoter. However, not only the magnitude of *SEPT2 *under-expression far exceeds the maximum 50% reduction that would be expected if one of the *SEPT2 *gene copies has its expression shutdown, but wild-type *MLL *expression seems to be strongly down-regulated as well. This suggests a concomitant down-regulation of wild-type *MLL*, wild-type *SEPT2*, and the *MLL-SEPT2 *fusion gene. The fact that *MLL*, *SEPT2*, and *MLL-SEPT2 *map to distinct chromosomes excludes a localized transcriptional repression affecting contiguous genes via a long-range control element as a possible mechanism.

Interestingly, *MLL *expression was also down-regulated in the group of *MLL *fusions other than *MLL-SEPT2*, when compared with the normal control group. Wild-type *MLL *down-regulation associated with *MLL *abnormalities was previously observed in AML with *MLL *partial tandem duplication (PTD) [27]. In that instance, the wild-type *MLL *transcript derived from the non-rearranged *MLL *allele was absent in the majority of cases of *MLL*-PTD, with the authors suggesting that the silencing of wild-type *MLL *may result from the action of the MLL-PTD protein via an auto-regulatory mechanism [27], which has so far not been described for *MLL-SEPT2*. In addition, down-regulation of *MLL *when fused with a partner gene was also previously observed in *MLL-MLLT3 *patients [27], suggesting that this can be a common event in *MLL*-related leukemia. Since MLL fusion proteins seem to transform by a gain-of-function mechanism with conversion of the MLL chimera into a potent transcriptional activator [28,29], quantitative oscillations in wild-type and chimeric *MLL *expression level presumably do not abrogate the leukemogenic properties of *MLL *fusion proteins. One alternative explanation for the observed down-regulation of *MLL*, *SEPT2 *and *MLL-SEPT2 *is the involvement of a transcriptional rather than post-transcriptional mechanism, for instance regulated via an epigenetic mark. DNA methylation within the promoter region of a gene can result in chromatin compaction and inhibition or down-regulation of gene transcription, and aberrant promoter methylation in cis is often responsible for gene silencing in a variety of malignancies [30]. However, the absence of DNA methylation in the CpG island located 5' of the *SEPT2 *gene (encompassing the predicted *SEPT2 *promoter region) suggests that hypermethylation of wild-type *SEPT2 *is probably not the mechanism responsible for the observed gene silencing but, since in only one *MLL-SEPT2 *case DNA was available to perform methylation analysis, a definitive conclusion cannot be draw.

How can *SEPT2 *down-regulation be associated with leukemogenesis? Mammalian septins have been linked with two distinct steps in cell division, namely during chromosome segregation and during cytokinesis, as depletion of septins by siRNA result in defects in both of these processes [31,32]. SEPT2 function is dependent of the formation of core oligomeric complexes with SEPT6 and SEPT7, and this septin heterotrimer is a recognized regulator of microtubule stability, with septin depletion resulting in a marked stabilization of microtubules and mitotic defects *in vivo *[1,33]. It is known that proper organization of the cytoskeleton, including that of septin filaments, is required for cell-cycle progression and, as a consequence, septins are indirectly involved in driving or halting the cell-cycle engine [34]. In addition, the SEPT2-SEPT6-SEPT7 complex can directly regulate cell-cycle progression by sequestering key signaling molecules involved in the DNA damage response and cell-cycle progression [33,34]. Down-regulation of the expression of septin genes has been described previously in neoplasia. Expression of the mitochondrial ARTS protein, a splice variant of the *SEPT4 *gene, is lost in the majority of childhood acute lymphoblastic leukemias and *SEPT9 *expression is down-regulated by promoter methylation in head and neck squamous cell carcinomas, suggesting that both genes can function as tumor suppressor genes [35,36]. *SEPT2 *may also play a role in the pathogenesis of other AML subtypes in which it is not involved as fusion partner, since we have uncovered significant *SEPT2 *RNA down-regulation in AML associated with the *PML-RARA *rearrangement and in AML with a normal karyotype.

*SEPT2 *is not the only septin family gene associated with hematological neoplasia. It has been shown that at least four other septins, *SEPT5*, *SEPT6*, *SEPT9 *and *SEPT11*, are also *MLL *fusion partners [9-13]. There is increasing evidence supporting the hypothesis that *MLL *fusion partners are not randomly chosen, but rather functionally selected. For instance, the most frequent MLL fusion partners AFF1 (AF4), MLLT3 (AF9), MLLT1 (ENL) and MLLT10 (AF10) have been shown to belong to the same nuclear protein network [37]. Furthermore, the carboxyl-terminal domain of *ELL *and *MLLT10 *have been shown to be required for the leukemic transformation associated with the MLL-ELL and MLL-MLLT10 fusion proteins, respectively [38,39].

## Conclusion

We provide evidence of *MLL *and *SEPT2 *down-regulation in *MLL-SEPT2 *myeloid neoplasia, as well as *MLL *under-expression in AML with *MLL *fusions with other partners other than *SEPT2 *but, due to the small number of *MLL-SEPT2 *cases available, these results should be confirmed in a larger series of patients. Characterization of the expression profile of other *MLL *fusion partners, including other septins, in hematological malignancies may allow a better understanding of the pathobiological mechanisms of AML.

## Competing interests

The authors declare that they have no competing interests.

## Authors' contributions

NC designed and performed the research, analyzed the data and drafted the manuscript. JS performed the research and analyzed the data. SB performed the RT-PCR studies. VC performed the qMSP studies. FRR analyzed the data. SL, CC, LT, and JV performed the chromosome banding and molecular cytogenetic studies. SS, CHM, and AB characterized patients 60 and 61 and provide samples of both patients for RT-PCR and qRT-PCR studies. JMM and LN clinically assessed the patients. MRT coordinated the study and participated in manuscript writing. All authors read and approved the final manuscript.

## Pre-publication history

The pre-publication history for this paper can be accessed here:

http://www.biomedcentral.com/1471-2407/9/147/prepub

## Supplementary Material

Additional file 1 **Additional Table S1**. Summary of clinical, molecular, and cytogenetic data of the 61 patients with hematological malignacies. Click here for file

Additional file 2 **Additional Table S2**. Oligonucleotide primers and probes (5'FAM, 3'TAMRA) used in this study. Click here for file
