# Supplementary material for: Both SEPT2 and MLL are down-regulated in MLL-SEPT2 therapy-related myeloid neoplasia
Source: BMC Cancer. 2009 May 15;9:147. doi: 10.1186/1471-2407-9-147 (PMC2689242; doi:10.1186/1471-2407-9-147)
Supplement: Additional file 1 — Additional Table S1. Summary of clinical, molecular, and cytogenetic data of the 61 patients with hematological malignacies. [file 1471-2407-9-147-S1.doc]

**Additional Table 1: Summary of clinical, molecular, and cytogenetic data of the 61 patients with hematological malignancies**.

| Patient | Age  (years) | Sex | FAB |  | Fusion Transcript | FISH | Karyotype |
| --- | --- | --- | --- | --- | --- | --- | --- |
| 1 | 55 | M | AML-M4 |  | *CBFB-MYH11* type A | *CBFB* | NM |
| 2 | 58 | F | AML-M4 |  | *CBFB-MYH11* type A | ND | 46,XX,inv(16)(p13q22)[6]/47,idem,+21[24] |
| 3 | 6 | M | AML-M4 |  | *CBFB-MYH11* type A | ND | 47,XY,inv(16)(p13q22),+22[8] |
| 4 | 53 | F | AML-M4 |  | *CBFB-MYH11* type A | ND | 46,XX,inv(16)(p13q22)[4]/46,XX[19] |
| 5 | 20 | M | AML-M4 |  | *CBFB-MYH11* type A | ND | 46,XY,t(5;17)(q13;q12),del(7)(q32),inv(16)(p13q22)[30] |
| 6 | 36 | F | AML-M4 |  | *CBFB-MYH11* type A | *CBFB* | 47,XX,+8,inv(16)(p13q22)[10] |
| 7 | 60 | F | AML-M4 |  | *CBFB-MYH11* type A | ND | 48,XX,+8,inv(16)(p13q22),+22[8]/46,XX[3] |
| 8 | 54 | F | AML-M4 |  | *CBFB-MYH11* type A | ND | 47,XX,inv(16)(p13q22),+22[30] |
| 9 | 16 | F | AML-M4 |  | *CBFB-MYH11* type A | *CBFB* | 46,XX,inv(16)(p13q22)[27]/46,XX[3] |
| 10 | 32 | F | AML-M4 |  | *CBFB-MYH11* type A | ND | 46,XX,inv(16)(p13q22)[20] |
| 11 | 67 | M | AML-M4 |  | *CBFB-MYH11* type A | ND | 46,XY,inv(16)(p13q22)[15] |
| 12 | 45 | F | AML-M4 |  | *CBFB-MYH11* type E | ND | 46,XX,inv(16)(p13q22)[28]/46,XX[2] |
| 13 | 41 | M | AML-M4 |  | *CBFB-MYH11* type E | ND | 46,XY,inv(16)(p13q22)[22] |
| 14 | 10 | F | AML-M3 |  | *PML-RARA* bcr 1 | ND | 46,XX,t(15;17)(q22;q21)[15]/46,XX[2] |
| 15 | 62 | M | AML-M3 |  | *PML-RARA* bcr 1 | N | 46,XY[30] |
| 16 | 43 | F | AML-M3 |  | *PML-RARA* bcr 1 | *PML-RARA* | 46,XX,t(15;17)(q22;q21)[3]/46,XX[14] |
| 17 | 11 | M | AML-M3 |  | *PML-RARA* bcr 1 | *PML-RARA* | 46,XY,t(6;19)(p21;q13),t(15;17)(q22;q21)[19] |
| 18 | 41 | M | AML-M3 |  | *PML-RARA* bcr 1 | *PML-RARA* | 46,XY,t(15;17)(q22;q21)[16]/46,XY[4] |
| 19 | 33 | M | AML-M3 |  | *PML-RARA* bcr 1 | *PML-RARA* | NM |
| 20 | 63 | M | AML-M3 |  | *PML-RARA* bcr 3 | *PML-RARA* | NM |
| 21 | 29 | M | AML-M3 |  | *PML-RARA* bcr 3 | ND | 47,XY,add(2)(p25),+8,t(15;17)(q22;q21)[23] |
| 22 | 29 | M | AML-M3 |  | *PML-RARA* bcr 3 | ND | 46,XY,t(15;17)(q22;q21)[12]/46,XY[2] |
| 23 | 55 | M | AML-M3 |  | *PML-RARA* bcr 3 | *PML-RARA* | 46,XY,t(15;17)(q22;q21)[30] |
| 24 | 45 | F | AML-M3 |  | *PML-RARA* bcr 3 | *PML-RARA* | 46,XX,add(12)(p12),t(15;17)(q22;q21)[3]/46,XX[14] |
| 25 | 49 | M | AML-M3 |  | *PML-RARA* bcr 3 | *PML-RARA* | 46,XY,t(15;17)(q22;q21)[18]/46,XY[2] |
| 26 | 44 | M | AML-M2 |  | RUNX1-RUNX1T1 | ND | 46,XY,t(8;21)(q22;q22)[11] |
| 27 | 33 | F | AML-M2 |  | *RUNX1-RUNX1T1* | ND | 46,XX,t(8;21)(q22;q22)[27]/46,XX[3] |
| 28 | 57 | F | AML-M2 |  | *RUNX1-RUNX1T1* | ND | 46,XX,t(8;21)(q22;q22)[28]/46,XX[2] |
| 29 | 46 | F | AML-M2 |  | *RUNX1-RUNX1T1* | ND | 46,XX,t(8;21)(q22;q22),del(9)(q22q32)[28]/46,XX[2] |
| 30 | 8 | M | AML-M2 |  | *RUNX1-RUNX1T1* | ND | 45,X,-Y,t(8;21)(q22;q22)[30] |
| 31 | 22 | F | AML-M2 |  | *RUNX1-RUNX1T1* | ND | 46,XX,del(7)(q32),t(8;21)(q22;q22)[16]/46,XX[14] |
| 32 | 43 | F | AML-M2 |  | *RUNX1-RUNX1T1* | ND | 46,XX,t(8;21)(q22;q22)[5]/46,idem,add(X)(p22)[15] |
| 33 | 75 | M | AML-M2 |  | *RUNX1-RUNX1T1* | ND | 46,XY,t(8;21)(q22;q22)[27]/46,XY[3] |
| 34 | 46 | F | AML-M2 |  | *RUNX1-RUNX1T1* | ND | 45,X,-X,t(8;21)(q22;q22)[19]/46,XX[11] |
| 35 | 41 | F | AML-M2 |  | *RUNX1-RUNX1T1* | ND | 46,XX,t(8;21)(q22;q22)[11] |
| 36 | 7 | M | AML-M2 |  | *RUNX1-RUNX1T1* | *RUNX1-RUNX1T1* | 45,X,-Y,t(8;21)(q22;q22)[17]/46,XY[3] |
| 37 | 66 | F | AML-M2 |  | *RUNX1-RUNX1T1* | ND | 45,X,-X,-2,add(7)(q31),t(8;21)(q22;q22),+mar[6]/46,XX[6] |
| 38 | 49 | F | AML-M5 |  | Normal Karyotype | N | 46,XX[30] |
| 39 | 64 | M | AML-M1 |  | Normal Karyotype | N | 46,XY[30] |
| 40 | 40 | F | AML-M1 |  | Normal Karyotype | N | 46,XX[20] |
| 41 | 62 | M | AML-M4 |  | Normal Karyotype | N | 46,XY[20] |
| 42 | 45 | F | AML-M5 |  | Normal Karyotype | N | 46,XX[20] |
| 43 | 58 | M | AML-M5 |  | Normal Karyotype | N | 46,XY[20] |
| 44 | 65 | M | AML-M0 |  | Normal Karyotype | N | 46,XY[20] |
| 45 | 36 | M | AML-M1 |  | Normal Karyotype | N | 46,XY[20] |
| 46 | 70 | F | AML-M2 |  | Normal Karyotype | N | 46,XX[10] |
| 47 | 52 | F | AML-M2 |  | Normal Karyotype | N | 46,XX[20] |
| 48 | 64 | F | AML-M2 |  | Normal Karyotype | N | 46,XX[20] |
| 49 | 40 | F | AML-M5 |  | *MLL-MLLT3* | MLL | 46,XX,t(9;11)(p22;q23)[14]/46,XX[16] |
| 50 | 1 | M | AML |  | *MLL-MLLT3* | *MLL* | 46,XY,t(9;11)(p22;q23)[23]/46,idem,add(1)(p22)[4]/46,XY[3] |
| 51 | 53 | F | AML-M5 |  | *MLL-MLLT3* | *MLL* | ND |
| 52 | 51 | M | AML-M5 |  | *MLL-MLLT3* | *MLL* | NM |
| 53 | 39 | F | AML-M5 |  | *MLL-MLLT3* | *MLL* | 46,XX,t(9;11)(p22;q23)[30] |
| 54 | 28 | M | AML-M5 |  | *MLL-ELL* | *MLL* | NM |
| 55 | 47 | F | AML-M5 |  | *MLL-ELL* | *MLL* | 46,XX,t(11;19)(q23;p13.1)[22] |
| 56 | 68 | M | AML-M5 |  | *MLL-MLLT1* | *MLL* | 47,XY,der(2)t(1;2)(q21;q37),+i(8)(q10),t(11;19)(q23;p13)[28]/47,idem,+8,  -i(8)(q10)[3] |
| 57 | 14 | M | AML-M1 |  | *MLL-MLLT10* | *MLL* | 46,XY,t(1;6)(p32;p22),t(3;22)(q13.3;q12),t(10;11)(p12;q23)[18].ish der(10)inv(10)(p12p12)t(10;11)(p12;q23)(5’MLL+,3’MLL+),  der(11)t(10;11)(p12;q23)( 5’MLL-,3’MLL-)[8] |
| 58 | 32 | M | AML-M4 |  | *MLL-MLLT11* | *MLL* | 46,XY,t(1;11)(q21;q23)[18]/46,XY[4] |
| 59 | 54 | F | AML-M4 |  | *MLL-SEPT2* | MLL | 46,XX,t(2;11)(q37;q23)[30] |
| 60 | 68 | M | AML-M2 |  | *MLL-SEPT2* | *MLL* | 46,XY,t(2;11)(q37;q23)[4]/51,idem,+8,+17,+21,+22,+mar[10]/46,XY[5] |
| 61 | 56 | F | MDS |  | *MLL-SEPT2* | *MLL* | 46,X,del(X)(q22q28),t(2;11)(q37;q23),del(7)(q22q36)[12]/46,XX[9] |

(ND) Not done; (N) Negative; (NM) No metaphases.
