# Supplementary material for: Both SEPT2 and MLL are down-regulated in MLL-SEPT2 therapy-related myeloid neoplasia
Source: BMC Cancer. 2009 May 15;9:147. doi: 10.1186/1471-2407-9-147 (PMC2689242; doi:10.1186/1471-2407-9-147)
Supplement: Additional file 2 — Additional Table S2. Oligonucleotide primers and probes (5'FAM, 3'TAMRA) used in this study. [file 1471-2407-9-147-S2.doc]

**Additional Table 2: Oligonucleotide primers and probes (5’FAM, 3’TAMRA) used in this study.**

The GenBank accession numbers for *MLL*, *SEPT2*, and *ABL1* are NM_005933.2, NM_001008491.1, and NM_005157, respectively.

| Gene | Exon | Position | Primer/Probe | Sequence 5’-3’ |
| --- | --- | --- | --- | --- |
| *MLL* | 4 | 3313_3336 | MLL-S | CATGGGAAGAACGAGAAAAGATTT |
| *MLL* | 5 | 3358_3382 | MLL-PR | ACAAGTCATCAATTGCTGGCTCAGA |
| *MLL* | 5 | 3385_3405 | MLL-AS | GTGGAGCAAGAGGTTCAGCAT |
| *SEPT2* | 3 | 503_525 | SEPT2-S | CACCGAAAATCAGTGAAAAAAGG |
| *SEPT2* | 3 | 528_554 | SEPT2-PR | TTGAGTTCACACTGATGGTGGTCGGTG |
| *SEPT2* | 4 | 565_589 | SEPT2-AS | GCTGTTTATGAGAGTCGATTTTCCT |
| *ABL1* | 2 | 225_255 | ABL1-S | TGGAGATAACACTCTAAGCATAACTAAAGGT |
| *ABL1* | 3 | 293_320 | ABL1-PR | CCATTTTTGGTTTGGGCTTCACACCATT |
| ABL1 | 3 | 328_348 | ABL1-AS | GATGTAGTTGCTTGGGACCCA |
| SEPT2 CpG | - | -9277_-9258* | SEPT2CPG-S | AGGAAAGGATATTTCGGGTC |
| SEPT2 CpG | - | -9244_-9224* | SEPT2CPG-PR | CGGGTCGGAGTCGTTCGCGTT |
| SEPT2 CpG | - | -9179_-9160* | SEPT2CPG-AS | CACGAACAACGACGTAACTC |
| ACTB | - | -1713_-1689** | ACTB-S | TGGTGATGGAGGAGGTTTAGTAAGT |
| ACTB | - | -1671_-1642** | ACTB-PR | ACCACCACCCAACACACAATAACAAACACA |
| ACTB | - | -1607-1581** | ACTB-AS | AACCAATAAAACCTACTCCTCCCTTAA |

(*) Position relative to the *SEPT2* gene transcriptional initiation site; (**) Position relative to the *ACTB* gene transcriptional initiation site; (S) Sense; (AS) Antisense; (PR) Probe.
